# Rapamycin Suppresses Penile NADPH Oxidase Activity to Preserve Erectile Function in Mice Fed a Western Diet

**DOI:** 10.3390/biomedicines10010068

**Published:** 2021-12-30

**Authors:** Justin D. La Favor, Clifford J. Pierre, Trinity J. Bivalacqua, Arthur L. Burnett

**Affiliations:** 1 Department of Nutrition and Integrative Physiology, Florida State University, Tallahassee, FL 32306, USA; cp15f@my.fsu.edu; 2 Department of Urology, The James Buchanan Brady Urological Institute, The Johns Hopkins School of Medicine, Baltimore, MD 21287, USA; Trinity.Bivalacqua@Pennmedicine.upenn.edu (T.J.B.); aburnet1@jhmi.edu (A.L.B.)

**Keywords:** erectile dysfunction, Nox2, mTOR, p70S6K, microdialysis, high-fat, high-sugar, ROS, oxidative stress, obesity

## Abstract

The mechanistic target of rapamycin (mTOR) is a nutrient-sensitive cellular signaling kinase that has been implicated in the excess production of reactive oxygen species (ROS). NADPH oxidase-derived ROS have been implicated in erectile dysfunction pathogenesis. The objective of this study was to determine if mTOR is an activator of NADPH oxidase in the penis and to determine the functional relevance of this pathway in a translationally relevant model of diet-induced erectile dysfunction. Male mice were fed a control diet or a high-fat, high-sucrose Western style diet (WD) for 12 weeks and treated with vehicle or rapamycin for the final 4 weeks of the dietary intervention. Following the intervention, erectile function was assessed by cavernous nerve-stimulated intracavernous pressure measurement, in vivo ROS production was measured in the penis using a microdialysis approach, and relative protein contents from the corpus cavernosum were determined by Western blot. Erectile function was impaired in vehicle treated WD-mice and was preserved in rapamycin treated WD-mice. Penile NADPH oxidase-mediated ROS were elevated in WD-mice and suppressed by rapamycin treatment. Western blot analysis suggests mTOR activation with WD by increased active site phosphorylation of mTOR and p70S6K, and increased expression of NADPH oxidase subunits, all of which were suppressed by rapamycin. These data suggest that mTOR is an upstream mediator of NADPH oxidase in the corpus cavernosum in response to a chronic Western diet, which has an adverse effect on erectile function.

## 1. Introduction

There has been a steady decline in the amount of time that Americans spend preparing their own food over the past six decades, a trend that spans all socioeconomic classes [1]. This trend has led to a steady incline in the consumption of processed, packaged, and pre-prepared foods, which are often replete with added sugar and fat from cheap vegetable oils [2]. This dietary pattern has been termed the Western diet due to its popularity in North America and Western Europe [3]. Increasing prevalence of the Western diet is associated with increased obesity rates and detrimental effects on cardiometabolic health [4,5,6,7]. Additionally, epidemiological studies indicate that Western style diets also increase risk for erectile dysfunction (ED) [8,9]. This is of concern as recent dietary trends and projections suggest an increased consumption of ultraprocessed foods, sugar-sweetened foods and beverages, and vegetable oils in many regions of the world not traditionally associated with Western society [10]. Organic ED is a prevalent condition amongst men with obesity, which may develop in response to one or a combination of vascular, endocrine, and neurologic deficiencies [11]. Due to the multifactorial nature of the condition, challenges remain regarding efficacy assessment of interventional treatments and optimization of therapeutic approach [11]. As ED often precedes the development of cardiovascular disease, further understanding of the pathophysiology of ED in response to obesogenic environments may provide insights for advances in men’s health.

We have previously developed a rodent Western diet that is reflective of these increasingly prevalent dietary patterns. This diet is high in sugar and high in fat, with a fatty acid distribution that would be expected from a diet largely derived from fast foods and processed foods [12]. Young adult rats fed this Western diet have been found to develop impaired erectile function after 8 weeks of the diet [12]. In a follow-up study, nerve-stimulated erectile function was acutely improved by intracavernous injection of an NADPH oxidase (Nox) inhibitor in young adult rats fed the Western diet for 12 weeks [13]. These findings implicate a role for Nox-derived reactive oxygen species (ROS) in the impaired erectile response. However, it is unclear what mechanisms provide the stimulus for elevated Nox activity in the corpus cavernosum in response to the Western diet intervention. 

The mechanistic target of rapamycin (mTOR) is a major cellular signaling kinase that exists in two complexes, complex 1 (mTORC1), which is highly sensitive to rapamycin, and complex 2 (mTORC2), which is minimally sensitive to rapamycin [14]. mTORC1 is particularly sensitive to nutrients, such as fatty acids and glucose, which tightly couples mTORC1 activity to the energy sensing pathways [15]. Indeed, mTORC1 signaling has been found to be upregulated in adipocytes [16], liver [17,18], kidney [19], heart [20], and skeletal muscle [17] of mice fed a high-fat diet. Additionally, exposure of cardiac myocytes to high-glucose [21], endothelial cells to high-glucose [22], and endothelial cells to high-fat [23] conditions induces mTORC1 signaling and increased ROS production. Inhibition of mTORC1 signaling by rapamycin treatment [21] or silencing of p70S6K [23], the downstream mediator of mTORC1 signaling blunted the elevation in ROS production induced by nutrient overload, implicating mTORC1 as a nutrient-sensitive mediator of ROS induction in endothelial cells and the cardiovascular system. We therefore hypothesize that mTORC1 may be an important node for the induction of ROS that is in part responsible for the pathogenesis of diet-induced erectile dysfunction.

The aim of this study was to determine if mTORC1 is an upstream activator of Nox in the penis in response to the Western diet and to determine if this pathway is relevant in the development of Western diet-induced ED.

## 2. Materials and Methods

### 2.1. Experimental Animals and Dietary Intervention

Male C57Bl/6J mice were purchased from Jackson Laboratories (Bar Harbor, ME, USA). Mice were maintained in a pathogen-free animal housing facility with ad libitum access to food and drinking water. Twelve-week dietary interventions were initiated at 8 weeks of age, such that all terminal procedures were performed at 20–21 weeks of age. Mice were fed either a high-fat, high-sucrose diet with a Western pattern fatty acid distribution (Teklad Diets 110365; Harlan Laboratories, Madison, WI, USA) (WD) or a control diet (Teklad Diets 110367) (CD). The WD (4.68 kcal/g, 44.6% kcal fat, 40.7% kcal carbohydrate, 340 g/kg sucrose, 27 ω-6:ω-3 ratio), and CD (3.67 kcal/g, 12.7% kcal fat, 72.4% kcal carbohydrate, 150 g/kg sucrose, 9.6 ω-6:ω-3 ratio) contained equivalent levels of vitamins, minerals, and protein when considered on a basis of energy density, as further described [12]. All experimental procedures were approved by the Institutional Animal Care and Use Committee of the Johns Hopkins University School of Medicine (protocol #MO18M206).

### 2.2. Rapamycin Treatment

Mice in each dietary group were designated for either vehicle or rapamycin treatment groups. Mice received an intraperitoneal injection of vehicle, 1 mg/kg rapamycin (LC Labs, Woburn, MA, USA), or 2 mg/kg rapamycin, on three nonconsecutive days for the final four weeks of the dietary intervention. Rapamycin solutions were prepared in a mixture of 60% saline, 30% ethanol, and 10% dimethyl sulfoxide [24]. Prior to administration, rapamycin and vehicle solutions were passed through a sterile filter and stored in a sterile microcentrifuge tube. Intraperitoneal injections were administered at a volume of 0.25 mL/kg using a sterile insulin syringe with ultrafine 30G needle (Becton Dickinson #328466, Franklin Lakes, NJ, USA). Body weights were measured prior to each administration with a digital electronic scale (Ohaus Corp., Parsippany, NJ, USA) to determine the volume of injection.

### 2.3. Metabolic Parameters and Anthropometrics

Lean mass and fat mass were measured at the end of the dietary/treatment intervention by nuclear magnetic resonance–magnetic resonance imaging (EchoMRI 100; Echo Medical Systems, Houston, TX, USA). Body fat percentage was calculated as (fat mass)/(body mass) × 100. Blood was obtained for glucose measurements by clipping the distal 1 mm of tail, allowing for glucose measurement from the second drop of blood produced using a glucometer (Accu-Chek, Roche Diagnostics, Basel, Switzerland). For fasting glucose measurements, all food was removed from the cage at 10:00 am, and a blood glucose reading was obtained at 4:00 pm. For nonfasting glucose measurement, a glucose reading was obtained at 10:00 am with no food restriction overnight. For blood lipid measurement, blood was obtained via cardiac puncture. Serum was separated using serum separator tubes and centrifugation at 14,000 rpm for 20 min. Serum was stored at −20 °C until analysis by a lipid panel for measurement of total cholesterol, triglycerides, high-density lipoprotein cholesterol (HDL-C), and low-density lipoprotein cholesterol (LDL-C) by the Johns Hopkins Molecular and Comparative Pathobiology phenotyping core facility.

### 2.4. Erectile Function Assessment

Mice were anesthetized with an intraperitoneal injection of 90 mg/kg ketamine and 10 mg/kg xylazine and supplemented as needed. Erectile function was assessed through measurement of intracavernosal pressure (ICP) and mean arterial pressure (MAP) as described previously [25]. The left carotid artery was cannulated with polyethylene (PE) tubing filled with 100 U/mL of heparinized saline connected to a pressure transducer, allowing for continuous measurement of MAP with LabChart7 software (ADInstruments, Sydney, Australia). The shaft of the penis was degloved, and a 30-gauge needle connected to heparinized saline filled-PE tubing was inserted into the crus. This cannula was connected to a pressure transducer, allowing for the continuous measurement of ICP with LabChart7 software. Platinum bipolar hook electrodes (Cat. #F-E2-48, Natus Neurology, Middleton, WI, USA) were placed underneath the right cavernous nerve, and were gently lifted upward using a micromanipulator (World Precision Instruments, Sarasota, FL, USA), which isolated the cavernous nerve from the prostate. The cavernous nerve was stimulated with a square pulse stimulator (Grass Instruments, Quincy, MA, USA) for 1 min stimulation periods, separately at 1, 2, and 4 V of stimulation. Simulation periods were separated by 5 min to allow for detumescence. Erectile function was assessed by the area under the curve (AUC) of the ICP pressure tracing during the stimulation period normalized to MAP (AUC/MAP), which is reflective of the ability to achieve and maintain an erection upon nervous stimulation. Mice were sacrificed upon completion of functional assessment.

### 2.5. Penile In Vivo ROS Measurement

Separate groups of mice from those that underwent testing for erectile function were used for assessment of penile in vivo ROS production. Only the 2 mg/kg rapamycin dose was used for these mice. Following completion of the dietary/treatment interventions, in vivo H_2_O_2_ and superoxide were measured using a microdialysis technique as previously described in detail [26,27]. Briefly, mice were anesthetized with an intraperitoneal injection of 90 mg/kg ketamine and 10 mg/kg xylazine and supplemented with an intramuscular injection as needed and placed on a small animal heated pad (Jorgensen Laboratories, Loveland, CO, USA) for the duration of the procedure. The penis was degloved, and a linear microdialysis probe with a 3 mm membrane length and 20-kDa maximal pore size (Bioanalytical Systems, Inc., West Lafayette, IN, USA) was implanted into the penile shaft. Microdialysis probes were perfused with saline containing 100 µM Amplex Ultrared (Molecular Probes, Eugene, OR, USA) and 1.0 U/mL horseradish peroxidase (HRP; Sigma Aldrich, St. Louis, MO, USA) at a rate of 1.0 µL/min. In the presence of HRP, Amplex Ultrared reacts with H_2_O_2_ to produce resorufin, a fluorescent byproduct. Three replicate dialysate samples were collected at 15-min intervals, and fluorescence was measured in a fluorometer (BioRad, Hercules, CA, USA) at 510 nm excitation and 590 nm emission. Following these measures, 10 U/mL superoxide dismutase (SOD, Sigma Aldrich) was added to the perfusate. Superoxide that diffuses into the microdialysis membrane may be converted into H_2_O_2_ by SOD, which further stimulates the Amplex Ultrared-HRP-resorufin reaction. Three replicate dialysate samples were collected at 15-min intervals from which fluorescence was measured. Then, 300 µM apocynin (Sigma Aldrich), an NADPH oxidase (Nox) inhibitor, was added to the Amplex Ultrared/HRP/SOD perfusate solution to test the impact of Nox activity on ROS production [28]. Apocynin was perfused for 30 min to allow for sufficient tissue penetrance, after which three 15-min replicate samples were collected, from which fluorescence was measured. The mice were sacrificed following these measurements.

### 2.6. Western Blotting

An additional group of mice underwent the dietary and vehicle or 2 mg/kg rapamycin treatment interventions in order to obtain naïve penile tissue for molecular analyses. Following the intervention, mice were anesthetized with an intraperitoneal injection of 90 mg/kg ketamine and 10 mg/kg xylazine. Penile tissue was exposed by freeing the tissue of skin and fascia. Mice were then sacrificed, and penile tissue from the base to the proximal glans was harvested, from which the corpus spongiosum, dorsal vein, and connective tissue were stripped off. The corpus cavernosum was quickly rinsed in ice-cold PBS, blood was removed from the tissue, and the tissue was snap frozen in liquid nitrogen and stored at −80 °C until processing. Each corpus cavernosum tissue sample was homogenized in 180 µL of radioimmunoprecipitation assay buffer (Cell Signaling Technologies, Danvers, MA, USA) with 1 mM phenylmethanesulfonyl fluoride (PMSF, Sigma Aldrich) in high strength glass tubes (Kimble Chase, Vineland, NJ, USA) on ice with a motor driven tissue homogenizer (Omni International, Kennesaw, GA, USA) with a stainless-steel generator probe (Omni International). Homogenate was centrifuged at 10,000× *g* for 30 min at 4 °C, and the soluble protein concentration was determined by the bicinchoninic acid assay (ThermoFisher Scientific, Waltham, MA, USA). Twenty-five micrograms of protein were loaded into each lane on 10% or 4–20% tris-glycine extended (TGX) stain-free precast Criterion gels (Biorad), separated by SDS-PAGE, and transferred to PVDF membranes (Genesee Scientific, San Diego, CA). Membranes were blocked with Superblock T20 Blocking Buffer (#37516, ThermoFisher Scientific) or 5% bovine serum albumin (BSA) in TBST. Membranes were probed overnight at 4 °C with primary antibodies, and then HRP conjugated anti-mouse (NA931, Cytiva Life Sciences, Marlborough, MA, USA) or anti-rabbit (NA934, Cytiva Life Sciences) secondary antibodies at room temperature for 1 h. Membranes were exposed to electrochemiluminescent detection reagent (Cytiva Life Sciences) and reactive bands were captured with a ChemiDoc imaging system (Biorad) and quantified by densitometry with Image J software (National Institutes of Health, Bethesda, MD, USA). Primary antibodies were obtained from Cell Signaling Technology (CST), BD Biosciences (BD), Santa Cruz Biotechnology (SC; Dallas, TX, USA), Protein Tech (PT; Rosemont, IL, USA), or ThermoFisher Scientific (TFS) and diluted in Superblock at the following dilutions: phospho-mTOR^Ser2448^ (CST #2971, 1:1000), mTOR (CST #2972, 1:1000), phospho-p70S6K^Thr389^ (CST #9205, 1:1000), p70S6K (CST #9202, 1:1000), gp91^phox^ (BD #611414, 1:1000), p67^phox^ (PT #15551-1-AP, 1:1000), p47^phox^ (SC #17845, 1:1000), p22^phox^ (SC #130550, 1:500), Nox4 (TFS #PA5-72816, 1:1000), and GAPDH (PT #10494-1-AP, 1:3000). Secondary antibodies were diluted 1:4000 in Superblock. Following imaging, membranes were stripped with Restore Western blot stripping buffer (TFS #21059) for 10 min, washed, and reprobed.

### 2.7. Data and Statistical Analysis

Statistical analyses were performed with GraphPad Prism (Prism 9.0, GraphPad, San Diego, CA, USA). Statistical differences for the voltage dependent erectile response were determined by two-way repeated measures ANOVA. All other measures were assessed by ordinary two-way ANOVA. Following ANOVA, Tukey’s multiple comparisons post hoc analysis was used to determine differences between individual groups. In all cases, *p* < 0.05 was considered statistically significant.

## 3. Results

### 3.1. Metabolic Parameters

Final body weights, body composition, blood glucose, and serum lipid levels are presented in Table 1. Mice fed the Western diet (WD) underwent an approximately 14% weight gain relative to control-diet-fed mice. Lean mass was similar among all groups, while fat mass and body fat percentage were doubled in mice fed the WD. Treatment with 2 mg/kg rapamycin thrice weekly for the final four weeks of the dietary interventions had no significant effects on body weight or body composition on mice exposed to either diet. A significant two-way ANOVA main effect (*p* = 0.002) of the WD was observed for fasting blood glucose, indicating an increase in fasting glucose following WD independent of rapamycin treatment status. However, a two-way ANOVA main effect (*p* = 0.006) of rapamycin treatment was observed for nonfasting glucose, suggesting that rapamycin suppresses glucose levels in the fed state. Both the WD and rapamycin treatment acted to increase total cholesterol (*p* < 0.001), LDL-cholesterol (*p* < 0.001), and HDL-cholesterol (*p* < 0.001), as significant two-way ANOVA main effects were observed for both conditions for all three cholesterol measures. Post hoc analysis revealed WD fed mice treated with rapamycin had higher total and LDL-cholesterol levels than all other groups. Aside from these differences in cholesterol, a two-way ANOVA main effect of the WD was observed for triglycerides, indicating a suppression of triglyceride levels in response to the WD.

### 3.2. Voltage-Dependent Erectile Response

**Figure 1:**
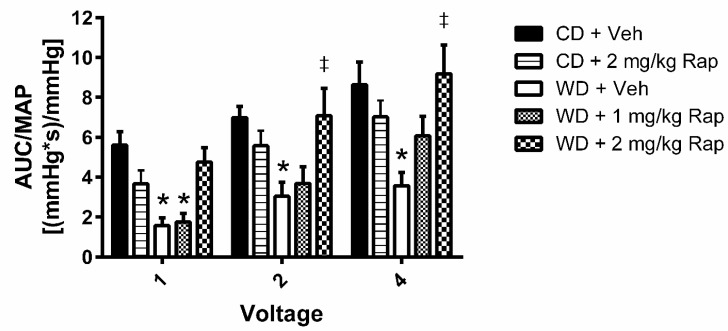
Assessment of cavernous-nerve-stimulated erectile function. Mice were fed the control (CD) or Western (WD) diet and treated with vehicle, 1 mg/kg rapamycin (Rap), or 2 mg/kg Rap. The cavernous nerve was stimulated at increasing voltages (1, 2, and 4 V) for one minute at each voltage. Erectile function is assessed by the area-under-the-curve (AUC) of the intracavernous pressure response to electrical stimulation normalized to mean arterial pressure (MAP). Data are presented as mean ± standard error of the mean for n = 7–10 mice per group; * *p* < 0.05 vs. CD + Veh, ‡ *p* < 0.05 vs. WD + Veh.

Cavernous-nerve-stimulated erectile function is presented in Figure 1. Erectile function was impaired at all voltages following 12 weeks of the Western diet in mice treated with vehicle injections (1 V: *p* = 0.008; 2 V: *p* = 0.010; 4 V: *p* < 0.001) relative to the corresponding control-diet-fed animals. Treatment of the Western-diet-fed mice with 1 mg/kg rapamycin yielded marginal effects on erectile function. However, treatment with 2 mg/kg rapamycin significantly augmented the erectile response at the 2 volt (*p* = 0.008) and 4 volt (*p* < 0.001) stimulation doses, restoring erectile function to similar levels as the control diet vehicle group. Treatment of the control diet animals with 2 mg/kg rapamycin induced a modest decrease in erectile function, although these differences did not reach statistical significance. 

### 3.3. The Western Diet Activates mTOR Signaling in the Corpus Cavernosum

**Figure 2:**
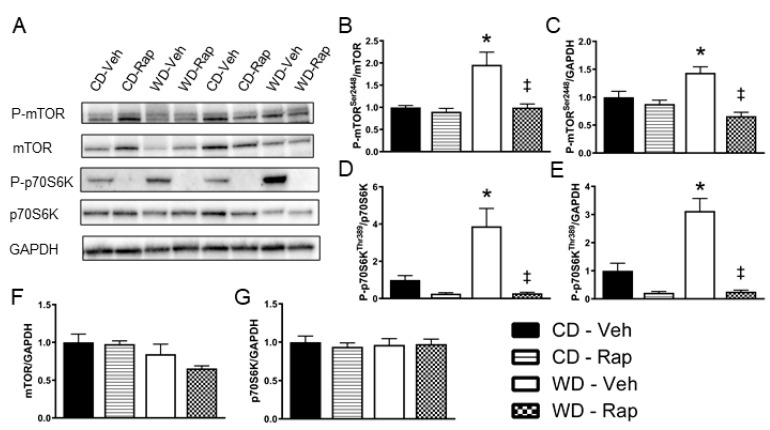
Assessment of mTOR signaling in the corpus cavernosum. Mice were fed the control (CD) or Western (WD) diet and treated with vehicle or 2 mg/kg rapamycin (Rap). (**A**) Representative immunoblot images for phosphorylated mTOR at Ser2448, total mTOR, phosphorylated p70S6K at Thr389, total p70S6K, and the loading control GAPDH. Quantification of band intensity density for: (**B**) phosphorylated mTOR normalized to total mTOR; (**C**) phosphorylated mTOR normalized to GAPDH; (**D**) phosphorylated p70S6K normalized to total p70S6K; (**E**) phosphorylated p70S6K normalized to GAPDH; (**F**) total mTOR normalized to GAPDH; (**G**) total p70S6K normalized to GAPDH. Data are presented as mean ± standard error of the mean for n = 8 mice per group; * *p* < 0.05 vs. CD-Veh, ‡ *p* < 0.05 vs. WD-Veh.

Representative images for Western blots ran against phosphorylated and total mTOR and p70S6K are presented in Figure 2A. Densitometry analysis revealed that phosphorylation of Serine 2448, the predominant activation site of mTOR, is increased (*p* = 0.008) in the corpus cavernosum of mice fed the Western diet. This response to the Western diet is blunted in mice treated with 2 mg/kg rapamycin, regardless of whether the densitometry expression is normalized to total mTOR content (*p* = 0.007, Figure 2B) or to the loading control of GAPDH (*p* < 0.001, Figure 2C). Similarly, active site phosphorylation (Threonine 389) of p70S6K, the major downstream effector of mTOR signaling, is upregulated (*p* = 0.015) in the corpus cavernosum of mice fed the Western diet. Again, this response to the Western diet is blunted in mice treated with 2 mg/kg rapamycin, regardless of whether the densitometry expression is normalized to total p70S6K content (*p* < 0.001, Figure 2D) or to GAPDH (*p* < 0.001, Figure 2E). Neither total protein contents of mTOR (Figure 2F) nor p70S6K (Figure 2G) were altered by the Western diet or rapamycin treatment.

### 3.4. Excessive Western Diet-Mediated Penile ROS Is Suppressed by Rapamycin

**Figure 3:**
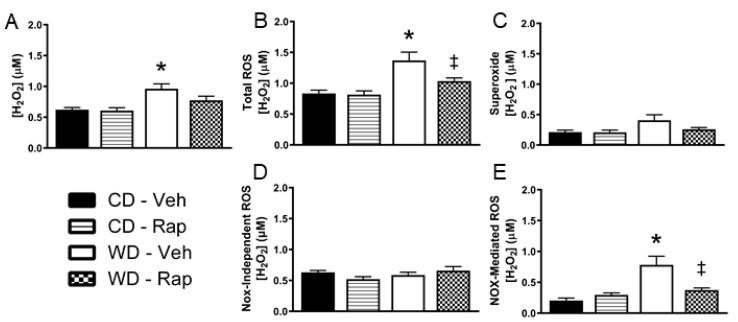
Assessment of in vivo penile reactive oxygen species (ROS) by microdialysis. Mice were fed the control (CD) or Western (WD) diet and treated with vehicle or 2 mg/kg rapamycin (Rap). (**A**) ROS were measured without superoxide dismutase (SOD) in the perfusate, indicative of H_2_O_2_ produced endogenously. (**B**) ROS were measured with SOD added to the perfusate, which is indicative of endogenous superoxide detected in addition to H_2_O_2_ (total ROS). (**C**) The H_2_O_2_ only signal was subtracted from the total ROS signal, reflecting the contribution of superoxide to the ROS measurement. (**D**) Total ROS were measured with the NADPH oxidase (Nox) inhibitor apocynin added to the perfusate. Nox-independent ROS were measured as the total ROS signal that was not inhibited by apocynin. (**E**) Nox-mediated ROS were determined by calculation of the ROS that was inhibited by apocynin. Data are presented as mean ± standard error of the mean for n = 9–10 mice per group; * *p* < 0.05 vs. CD-Veh, ‡ *p* < 0.05 vs. WD-Veh.

The Western diet increased in vivo penile H_2_O_2_ levels relative to the control diet under vehicle treated conditions (*p* = 0.014, Figure 3A). Addition of superoxide dismutase (SOD) to the microdialysis perfusate allows for additional contribution of superoxide that crosses into the microdialysis membrane to the total ROS fluorescent signal. Under these conditions, penile in vivo ROS levels were increased by the Western diet (*p* = 0.004), and significantly blunted by 2 mg/kg rapamycin treatment (*p* = 0.042, Figure 3B). The change in ROS signal upon addition of SOD to the microdialysis perfusate is presented in Figure 3C. There were no significant differences between groups for this measure, although there was a trend for increased superoxide for WD-Veh vs. CD-Veh mice (*p* = 0.067). Next, the NADPH oxidase (Nox) inhibitor apocynin was added to the microdialysis perfusate, allowing for apocynin penetrance into the interstitium of the local environment of the microdialysis membrane in the penis. The ROS detected with concomitant apocynin perfusion were not different between any of the groups (Figure 3D). The total ROS signal that was inhibited by apocynin was significantly increased by the Western diet (*p* = 0.001), which was blunted by rapamycin treatment (*p* = 0.006, Figure 3E). 

### 3.5. Western Diet-Induced Corpus Cavernosum NADPH Oxidase Protein Content Is Blunted by Rapamycin

**Figure 4:**
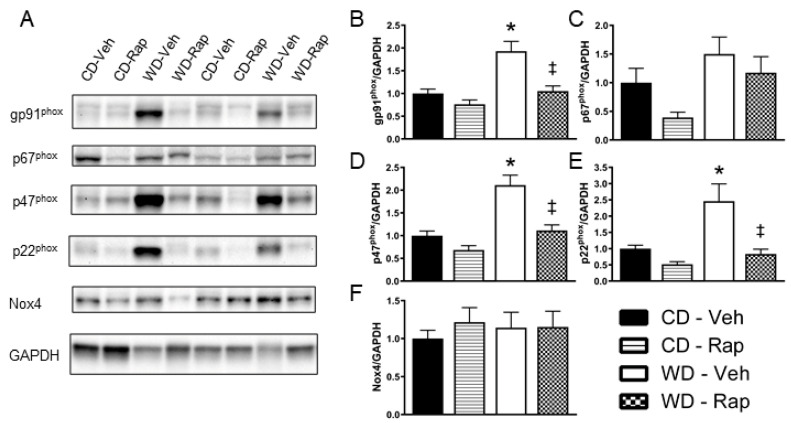
Assessment of NADPH oxidase subunit protein content in the corpus cavernosum. Mice were fed the control (CD) or Western (WD) diet and treated with vehicle or 2 mg/kg rapamycin (Rap). (**A**) Representative immunoblot images for the NADPH oxidase subunits and the loading control GAPDH. Quantification of band intensity density normalized to GAPDH for: (**B**) gp91^phox^; (**C**) p67^phox^; (**D**) p47^phox^; (**E**) p22^phox^; (**F**) Nox4. Data are presented as mean ± standard error of the mean for n = 8–9 mice per group; * *p* < 0.05 vs. CD-Veh, ‡ *p* < 0.05 vs. WD-Veh.

Representative images for Western blots ran against the Nox subunits gp91^phox^, p67^phox^, p47^phox^, p22^phox^, Nox4, and the loading control GAPDH in the corpus cavernosum are presented in Figure 4A. Relative protein content of gp91^phox^ (*p* = 0.004, Figure 4B), p47^phox^ (*p* < 0.001, Figure 4D), and p22^phox^ (*p* = 0.019, Figure 4E) were all significantly elevated by the Western diet, effects that were all significantly blunted by 2 mg/kg rapamycin treatment (gp91^phox^, *p* = 0.009; p47^phox^, *p* = 0.003; p22^phox^, *p* = 0.035, respectively). Relative content of p67^phox^ was increased in vehicle treated Western diet mice compared to rapamycin treated control diet mice (*p* = 0.016), although no other comparisons reached statistical significance (Figure 4C). There were no differences observed between any of the groups for Nox4 protein content (Figure 4F).

## 4. Discussion

In the present study, we investigated the efficacy of rapamycin treatment on erectile function in mice chronically fed a westernized diet that is high in sucrose and fat derived from saturated and ω-6 polyunsaturated fatty acids. We observed a restoration of erectile function with rapamycin treatment that was dose dependent. Furthermore, we observed an increase in mTORC1 signaling in the corpus cavernosum of the Western-diet-fed mice that was markedly suppressed by rapamycin treatment. Finally, rapamycin treatment blunted penile Nox-mediated ROS and protein expression of Nox2 subunits in the corpus cavernosum of mice fed the Western diet. 

mTORC1 is activated in response to insulin, growth factors, nutrients, and positive cellular energy status, through which mTORC1 acts as a key regulator of the cellular anabolic response [29]. One major pathway through which mTORC1 transmits these growth signals is the phosphorylation and activation of ribosomal protein S6 kinase (p70S6K) and subsequent promotion of mRNA translation and protein synthesis [29]. The mTORC1/p70S6K axis may serve as a nexus that connects overnutrition to accelerated cardiovascular aging and cardiovascular disease pathogenesis [30,31]. Indeed, overnutrition with a high-fat diet has been shown to increase active site phosphorylation of mTOR and p70S6K in the heart and aorta, while in vivo treatment with rapamycin blocks this pathway [32,33,34]. The present study demonstrates that chronic consumption of a Western style high-fat/high-sucrose diet induces elevated active site phosphorylation of mTOR and p70S6K in the corpus cavernosum, both of which were suppressed by rapamycin treatment. This finding extends the relevance of this pathway observed in cardiovascular diseases to ED.

Rapamycin has been used in two prior investigations of ED in rodent models [24,35]. In one study, rapamycin was shown to partially restore erectile function in a type 1 diabetic rat model [35]. Curiously, active site phosphorylation of mTOR and p70S6K was decreased in the corpus cavernosum of the diabetic rats relative to control rats, while 3 weeks of rapamycin treatment decreased active site phosphorylation even further [35]. Type 1 diabetes was induced during adolescence by administration of streptozotocin, after which rats lost significant weight despite initiation of diabetes at an age of rapid growth and physical development. It is plausible that corpus cavernosum mTORC1 signaling may be repressed in the absence of a chronic positive energy balance despite chronic hyperglycemia. Nevertheless, these investigators observed that further suppression of the mTORC1 pathway with rapamycin did suppress corporal fibrosis and had a restorative effect on neuronal and endothelial nitric oxide synthase content in the type 1 diabetic condition, suggesting a likely positive effect on vasodilatory pathways [35]. The other study demonstrated improvements in nerve-stimulated erectile function with rapamycin treatment both one day and seven days following bilateral cavernous nerve injury [24]. While mTOR signaling was not assessed, the principal molecular finding was that rapamycin-stimulated expression of the immunophilin protein FKBP12 in the major pelvic ganglion seven days following nerve injury, suggestive of a neuroprotective effect of rapamycin [24]. Collectively, these studies are in agreement with the findings of the present study, all of which demonstrated a restorative effect of rapamycin treatment on cavernous-nerve-stimulated erectile function. The nerve-stimulated erectile response used in these and the present study encompasses the summation of multiple events of the erection process, including neurovascular coupling, which is dependent on the integrity and function of the cavernous and penile nerves, the ability of internal iliac, internal pudendal, and penile arteries to dilate and funnel blood away from the systemic circulation for delivery to the penis, and the expansion of the corporal sinusoids [36]. While the duration and frequency of rapamycin treatment varies considerably across fields and within these studies, all studies that successfully improved erectile function used a 2 mg/kg dose for each administration. No significant functional effects were observed for the group treated with the 1 mg/kg dose in the present study, demonstrating an important impact of dosing for rapamycin treatment on the erectile system. 

Oxidative stress has been causatively linked to ED pathogenesis resulting from a variety of pathologies, including obesity, type 2 diabetes, and aging [37,38,39,40]. While the penile vasculature represents a unique circulatory system that is predominantly maintained in a low perfusion (flaccid) state primarily by α-adrenergic-mediated vasoconstriction [41], studies utilizing endothelial cell culture or arteries of the central circulation may provide insights into molecular functioning that may have potential relevance to ED pathology. Indeed, hyperactivation of the mTOR/p70S6K axis has been implicated in the excessive production of ROS in such models. Six weeks of dietary rapamycin supplementation to old mice has been found to attenuate ROS production, blunt hyperphosphorylation of the p70S6K active site, improve endothelium-dependent vasorelaxation, and decreases collagen deposition of the carotid artery that were all associated with aging [42]. The relevance of hyperactive p70S6K to endothelial aging has been further elucidated by Rajapakse et al. [43], who demonstrated that adenoviral transfection of constitutively active p70S6K increases superoxide, decreases NO production, and induces premature senescence in young endothelial cells. Conversely, silencing p70S6K reduces superoxide production and enhances NO production in senescent endothelial cells [43]. With relevance toward nutrient overload, incubation of cultured endothelial cells with rapamycin under high-glucose conditions reduced ROS generation, stimulated wound repair, and increased markers of angiogenic activation [44]. Similarly, exposure of cultured endothelial cells to a high dose of palmitic acid to simulate hyperlipidemic conditions induces an increase in ROS production that can be blunted by mTOR and p70S6K inhibitors, as well as by p70S6K siRNA [23]. Furthermore, continuous administration of these mTOR and p70S6K inhibitors throughout the course of a high-fat atherogenic diet intervention blunts oxidative stress and atherosclerotic lesions at the aortic root [23]. Collectively, these studies support the present study by demonstrating a mechanistic basis for mTOR/p70S6K induction of excess ROS production and subsequent impairment of endothelial NO production. As the nerve-stimulated erectile response is highly dependent on NO production [45], these pathways have relevance to ED pathophysiology. 

A recent study by Reho et al. [46] further implicated this system in endothelial ROS elevation by directly linking hyperactive mTORC1/p70S6K to Nox2 induction. They stimulated mTOR activity in cultured endothelial cells by exposure to a high concentration of leucine, which induced a 3.5-fold elevation in Nox2 mRNA expression. A 24-h high-leucine exposure to aortic rings in tissue culture resulted in an impairment in endothelium-dependent relaxation, which was prevented by adenoviral p70S6K knockdown. Conversely, adenoviral transfection of endothelial cells with constitutively active p70S6K resulted in a 3-fold increase in ROS production and a 4-fold increase in Nox2 gene expression over the control. Similarly, transfection of aortic rings with constitutively active p70S6K increased gp91^phox^ protein content and induced an impairment of endothelium-dependent relaxation, which was reversed by acute incubation with a superoxide dismutase mimetic, directly implicating the excess ROS induced by p70S6K activation in the functional impairment. In the present study, we observed an increase in protein content of gp91^phox^, the core catalytic subunit of Nox2, as well as the p22^phox^ and p47^phox^ subunits that combine to form the Nox2 complex, in the corpus cavernosum of Western-diet-fed mice. Further, these Western diet-induced increases in subunits of the Nox2 complex were suppressed by rapamycin treatment, suggesting that a hyperactive mTORC1 is in part responsible for these diet-mediated responses in the corpus cavernosum. 

Nox2 is a distinct target for ED, as subunits of the Nox2 complex have been found to be elevated in the corpus cavernosum in rodent models of atherosclerosis [47,48], diabetes [49,50], hypertension [51], and aging [52]. Apocynin is reported to inhibit the assembly of p47^phox^ and p67^phox^ within the membrane complex, and thus inhibits the Nox1 and Nox2 isoforms that require translocation of these subunits [53]. Nox2 has been causally implicated in the development of ED under pathological conditions, as chronic in vivo administration of apocynin preserves nerve-stimulated erectile function and suppresses protein content of Nox2 subunits in the corpus cavernosum [47,50,51]. Acute ex vivo apocynin exposure has also been found to improve nitrergic relaxation of penile arteries of obese Zucker rats [54] and corpus cavernosum of aging rats [52], while we have demonstrated that acute intracavernous injection of apocynin improves the nerve-stimulated erectile response in rats fed an identical Western diet intervention as that in the present study [13]. These studies that demonstrate functional improvement with acute apocynin exposure directly implicate Nox1/2 derived ROS in the impaired erectile process. As these functional measures are highly dependent on NO production, it is likely that the elevated Nox1/2 derived ROS in the erectile tissues of these models results in a reduction in NO bioavailability through quenching of NO by superoxide that results in the formation of peroxynitrite, and potentially in NOS uncoupling whereby the NOS enzyme produces superoxide rather than NO [47,54,55]. These acute effects of Nox1/2 inhibition are also relevant to situations in which Nox1/2 is chronically inhibited, while chronic Nox1/2 inhibition may inhibit the progressive development of fibrosis and vascular remodeling resulting from chronic oxidative stress.

In the present study, we measured penile H_2_O_2_ and superoxide in anesthetized mice using a microdialysis approach. We observed an approximately 65% increase in total ROS measured with this technique in the Western-diet-fed mice. This was primarily driven by increased H_2_O_2_, which was largely suppressed by chronic rapamycin treatment in the Western diet condition. We further utilized apocynin perfusion through the microdialysis probes to deliver apocynin to the local environment of the microdialysis membrane that was embedded in the penis. The suppression of total ROS signal upon apocynin perfusion is deemed Nox-mediated ROS, which was greater than 3-fold elevated in the penises of the Western diet-fed mice. This Western diet-induced increase in penile Nox-mediated ROS was largely blunted by chronic rapamycin treatment, which is reflective of the changes in protein expression of the Nox2 complex subunits. The proportion of the ROS signal that was not apocynin-inhibitable was deemed Nox-independent ROS and was not different amongst any of the groups, suggesting that the rapamycin-sensitive increase in penile ROS induced by the Western diet is primarily Nox1/2-mediated. The lack of difference in Nox4 protein content between any groups suggests that Nox4 is unlikely to contribute to excess penile ROS burden associated with the Western diet. It should be noted that microdialysis samples from the interstitium. Therefore, the ROS signal attained is not meant to reflect the total intracellular ROS levels, but rather is reflective of the ROS capable of cell–cell signal transduction [26,27]. The results from this study are in line with our prior investigation [28] in which we utilized a similar microdialysis approach to observe a greater than 4-fold elevation in Nox-mediated ROS in the skeletal muscle of obese human subjects without overt cardiometabolic disease.

While this study demonstrates a restoration in erectile function in the Western diet model with 4 weeks of rapamycin treatment, only a single duration of treatment was used. Future studies utilizing longer-term rapamycin treatment are needed to confirm a chronic benefit. In particular, we observed increases in serum total cholesterol, LDL-cholesterol, and HDL-cholesterol with rapamycin treatment regardless of dietary condition. While the increase in HDL-cholesterol may counteract the atherogenic effect of the increase in LDL-cholesterol, there may be adverse effects of this metabolic alteration on erectile function and overall health when extended to a longer duration. Rapamycin also has well-known immunosuppressive effects that may limit its translational potential for systemic use in treating human ED patients. Long-term use of systemic mTOR inhibitors as immunosuppressive agents in kidney transplant recipients is associated with reduced testosterone levels [56], which further limits the utility of chronic systemic mTOR inhibition in human ED populations. mTOR inhibitors have been approved for use in drug eluting vascular stents and balloons by regulatory agencies. These have recently been used in the pre-penile arteries for treatment of vascular ED with promising results [57,58], which may represent a feasible way to locally target drug delivery. Results from the present study provide proof-of-principle that the nutrient- and energy-sensitive mTORC1/p70S6K pathway contributes to the oxidative burden that is involved in ED pathogenesis in the corpus cavernosum. Many of the effects of caloric restriction on increased longevity have been attributed to a reduction in mTORC1 signaling [59]. With this in mind, caloric restriction and/or time restricted feeding may be explored as a possible means of improving erectile function in obese men with early stage ED or as a strategy to improve PDE-5 inhibitor efficacy, which may result in an overall cardiovascular benefit. Additionally, future investigations may address the effects of compounds that may suppress mTORC1 activity without the drastic immunosuppressive effects or hyperlipidemic effects of rapamycin, such as resveratrol [60]. Finally, future research should seek to tease apart individual nutrient contributions to mTORC1 activation in the corpus cavernosum, such as high-sugar vs. high-fat exposures, or exposures to high-fat diets of differing fatty acid composition to gain a more complete understanding of how nutrients impact this pathway, and ultimately to design more effective nutritional strategies for men concerned with erectile function. Nevertheless, this study offers significant translational value through use of a diet that has a mixture of these components in proportions that have become prevalently consumed in Western society.

## 5. Conclusions

The mTORC1/p70S6K axis appears to be an upstream mediator of Nox2 in the corpus cavernosum in response to chronic consumption of a high-fat, high-sucrose, Western pattern diet, which has a deleterious effect on erectile function. These findings have implications for the mechanistic basis through which poor nutrition practices influence ED pathophysiology.

## Figures and Tables

**Table 1:** Metabolic parameters.

	CD-Veh	CD-Rap	WD-Veh	WD-Rap
Body Weight (g)	27.6 ± 0.82	27.8 ± 0.55	31.6 ± 2.99 *†	31.5 ± 2.92 *†
Lean Mass (g)	22.3 ± 0.78	23.8 ± 0.58	22.6 ± 0.74	21.2 ± 0.39
Fat Mass (g)	3.42 ± 0.16	2.18 ± 0.13	6.56 ± 0.54 *†	7.58 ± 0.51 *†
Body Fat %	11.8 ± 0.62	10.6 ± 0.46	20.0 ± 1.48 *†	23.6 ± 1.38 *†
Fasting Glucose (mg/dL)	84.7 ± 7.52	93.8 ± 8.04	116 ± 8.30	118 ± 9.42 *
Nonfasting Glucose (mg/dL)	132 ± 12.8	94.4 ± 3.48	141 ± 12.1	119 ± 5.39 †
Total Cholesterol (mg/dL)	134 ± 4.2	196 ± 9.2 *	169 ± 16.4	248 ± 7.2 *†‡
Triglycerides (mg/dL)	105 ± 16 §	95.9 ± 9.6	75.2 ± 5.6	57.3 ± 3.0
LDL-Cholesterol (mg/dL)	57.8 ± 2.8	101 ± 6.1 *	104 ± 15.8 *	146 ± 4.4 *†‡
HDL-Cholesterol (mg/dL)	55.4 ± 2.0	75.7 ± 4.7 *	73.1 ± 3.3 *	90.6 ± 4.1 *‡

Data are presented as mean ± SEM for n = 10 per group; * *p* < 0.05 vs. CD-Veh, † *p* < 0.05 vs. CD-Rap, ‡ *p* < 0.05 vs. WD-Veh, § *p* < 0.05 vs. WD-Rap. All significance symbols indicate a value greater than the comparison group.

## Data Availability

Data are available upon reasonable request from the corresponding author.
